# Transcriptomics of type 1 diabetes progression: a validation study in newly diagnosed patients

**DOI:** 10.1016/j.ebiom.2026.106374

**Published:** 2026-07-07

**Authors:** Tomi Suomi, Inna Starskaia, Omid Rasool, Ubaid Ullah Kalim, Sylvaine Bruggraber, M. Loredana Marcovecchio, Emile Hendricks, Lut Overbergh, Mark Peakman, Timothy Tree, Søren Brunak, Anke M. Schulte, Chantal Mathieu, Mikael Knip, Riitta Lahesmaa, Laura L. Elo, INNODIA Consortium, C. Mathieu, P. Gillard, K. Casteels, L. Overbergh, D. Dunger, C. Wallace, M. Evans, A. Thankamony, E. Hendriks, S. Bruggraber, L. Marcovecchio, M. Peakman, T. Tree, N.G. Morgan, S. Richardson, R. Oram, J.A. Todd, L. Wicker, A. Mander, C. Dayan, M. Alhadj Ali, T. Pieber, D.L. Eizirik, M. Cnop, S. Brunak, F. Pociot, J. Johannesen, P. Rossing, C. Legido Quigley, R. Mallone, R. Scharfmann, C. Boitard, M. Knip, T. Otonkoski, R. Veijola, R. Lahesmaa, M. Oresic, J. Toppari, T. Danne, A.G. Ziegler, P. Achenbach, T. Rodriguez-Calvo, M. Solimena, E.E. Bonifacio, S. Speier, R. Holl, F. Dotta, F. Chiarelli, P. Marchetti, E. Bosi, S. Cianfarani, P. Ciampalini, C. De Beaufort, K. Dahl-Jørgensen, T. Skrivarhaug, G. Joner, L. Krogvold, P. Jarosz-Chobot, T. Battelino, B. Thorens, M. Gotthardt, B.O. Roep, T. Nikolic, A. Zaldumbide, A. Lernmark, M. Lundgren, G. Giordano, J.F. Tajes, G. Costecalde, T. Strube, A.M. Schulte, A. Nitsche, M. Peakman, J. Vela, M. Von Herrath, J. Wesley, A. Napolitano-Rosen, M. Thomas, N. Schloot, A. Goldfine, F. Waldron-Lynch, J. Kompa, A. Vedala, N. Hartmann, G. Nicolas, J. van Rampelbergh, N. Bovy, S. Dutta, J. Soderberg, S. Ahmed, F. Martin, E. Latres, G. Agiostratidou, A. Koralova, R. Willemsen, A. Smith, B. Anand, V. Datta, V. Puthi, S. Zac-Varghese, R. Dias, P. Sundaram, B. Vaidya, C. Patterson, K. Owen, C. Dayan, B. Piel, S. Heller, T. Randell, T. Gazis, E. Bismuth Reisman, J.-C. Carel, J.-P. Riveline, J.-F. Gautier, F. Andreelli, F. Travert, E. Cosson, A. Penfornis, C. Petit, B. Feve, N. Lucidarme, E. Cosson, J.-P. Beressi, C. Ajzenman, A. Radu, S. Greteau-Hamoumou, C. Bibal, T. Meissner, B. Heidtmann, S. Toni, B. Rami-Merhar, B. Eeckhout, B. Peene, N. Vantongerloo, T. Maes, L. Gommers

**Affiliations:** a Turku Bioscience Centre, University of Turku and Åbo Akademi University, FI-20520, Turku, Finland; b InFLAMES Research Flagship Center, University of Turku, Turku, Finland; c Department of Paediatrics, University of Cambridge, Cambridge, UK; d Department of Chronic Diseases and Metabolism, Endocrinology, Katholieke Universiteit Leuven, Leuven, Belgium; e Immunology & Inflammation Research Therapeutic Area, Sanofi, MA, USA; f Department of Immunobiology, King's College, London, UK; g Novo Nordisk Foundation Center for Protein Research, Faculty of Health and Medical Sciences, University of Copenhagen, Copenhagen, Denmark; h Sanofi-Aventis Deutschland GmbH, Frankfurt, Germany; i Paediatric Research Centre, University of Helsinki and Helsinki University Hospital, Helsinki, Finland; j Research Program for Clinical and Molecular Metabolism, Faculty of Medicine, University of 9 Helsinki, Helsinki, Finland; k Tampere Centre for Child Health Research, Tampere University Hospital, Tampere, Finland; l Institute of Biomedicine, University of Turku, FI-20520, Turku, Finland

**Keywords:** Type 1 diabetes, Transcriptomics, Beta cell decline, C-peptide, Disease progression

## Abstract

**Background:**

Type 1 diabetes is an autoimmune disease with significant long-term complications. Variability in the decline of insulin secretion after diagnosis complicates both the development of treatments and disease management. We previously reported that gene expression changes within the first year post-diagnosis were associated with C-peptide decline at two years in the first INNODIA cohort of patients with newly diagnosed type 1 diabetes. Here, we aimed to validate these findings in an independent follow-up cohort and to increase statistical power by combining the data from both cohorts.

**Methods:**

We analysed transcriptomic data from a follow-up INNODIA cohort of 168 individuals with newly diagnosed type 1 diabetes to assess whether previously identified associations with disease progression could be replicated. We then combined data from the original and follow-up cohorts for integrated analysis. Longitudinal gene expression changes during the first year after diagnosis were examined in relation to disease progression, alongside age and estimated immune cell abundances.

**Findings:**

Analysis of the follow-up cohort validated the previously observed longitudinal changes in gene expression during the first year after diagnosis. In the combined dataset, transcriptomic analysis identified a large number of genes that were differentially expressed during the first year after disease onset. More rapid disease progression was associated with younger age and a relative decrease in neutrophil abundance. In addition, changes in the expression of several genes were associated with the rate of disease progression.

**Interpretation:**

These findings support the existence of biological heterogeneity in disease progression after diagnosis of type 1 diabetes and contribute to an improved understanding of the molecular dynamics associated with disease progression. These findings may help future studies aiming to enable patient stratification and design of more targeted and personalised therapeutic approaches in type 1 diabetes.

**Funding:**

This project has received funding from the Innovative Medicines Initiative 2 Joint Undertaking under grant agreement No 115797 (INNODIA) and No 945268 (INNODIA HARVEST).

**Research in context**

**Evidence before this study**

Type 1 diabetes is characterised by autoimmune destruction of pancreatic β-cells, but the rate of post-diagnosis decline in insulin secretion varies between individuals. Circulating C-peptide reflects β-cell function, and several studies have demonstrated heterogeneity in C-peptide decline after disease onset. Transcriptomic profiling of peripheral blood has shown promise in identifying immune-related changes in type 1 diabetes, including interferon-associated signatures, yet many studies have been limited by small sample sizes, cross-sectional designs, or lack of validation. As part of the INNODIA consortium, we previously reported changes in peripheral blood gene expression during the first year after diagnosis in a cohort of 100 newly diagnosed patients, including associations with the rate of C-peptide decline at two years. However, validation in a larger cohort was required to establish the robustness and generalisability of these findings.

**Added value of this study**

This study provides validation of previously identified progression-associated transcriptomic changes in a follow-up cohort of 168 newly diagnosed patients with type 1 diabetes from the INNODIA study. By combining transcriptomic data from two cohorts, we substantially increased statistical power and enabled a more comprehensive characterisation of gene expression changes occurring during the first year after disease onset. We confirmed consistent expression patterns for the top genes previously associated with disease progression and identified numerous additional differentially expressed genes, including down-regulation of interferon-related pathways. We also identified associations between more rapid disease progression and younger age, reduced relative neutrophil abundance, and changes in the expression of several specific genes. These findings strengthen the evidence that early post-diagnosis blood transcriptomic changes reflect underlying disease heterogeneity and are associated with subsequent loss of β-cell function.

**Implications of all the available evidence**

The combined evidence indicates that the early period after type 1 diabetes diagnosis is characterised by dynamic changes in peripheral blood gene expression that are associated with the rate of disease progression. Blood-based transcriptomic signatures, particularly when validated across independent cohorts, have the potential to support patient stratification and risk prediction in individuals with newly diagnosed type 1 diabetes. These insights may inform the design of more efficient clinical trials and could contribute to the development of personalised therapeutic approaches aimed at preserving residual β-cell function.

## Introduction

Type 1 diabetes (T1D) is one of the most common chronic autoimmune diseases in children. It is characterised by immune-mediated destruction of insulin-producing pancreatic β-cells, leading to dependence on exogenous insulin. Despite advances in disease management, T1D is associated with severe long-term complications and presents a significant burden to the individuals living with T1D, their families, and health care systems.

Accumulating evidence indicates considerable heterogeneity in the rate of post-onset insulin secretion decline, which can be measured using circulating C-peptide as a marker of residual β-cell function. Heterogeneity in T1D is evident already during preclinical stages and across age at onset, immunopathology, residual insulin secretory capacity, rate of disease progression, and response to therapy, consistent with the existence of biologically distinct disease endotypes.^1^, ^2^, ^3^, ^4^ This heterogeneity is also evident after diagnosis, as the rate of C-peptide decline differs substantially between individuals and is linked to age-related immune phenotypes.^5^^,^^6^ In particular, younger individuals generally show a more rapid decline in insulin secretion and more aggressive immune profiles, whereas pancreatic studies have identified age-associated CD20Hi and CD20Lo patterns that differ in immune-cell infiltration and residual β-cell preservation.^5^, ^6^, ^7^ Preservation of C-peptide is clinically meaningful, as higher stimulated C-peptide levels are associated with lower insulin requirements, better glycaemic control, and reduced risk of hypoglycaemia and retinopathy.^8^ In addition, evidence from islet transplantation studies suggests that restoration of endogenous insulin secretion may provide broader clinical benefits beyond glycaemic control, including cardiovascular and other diabetes-related outcomes.^9^, ^10^, ^11^

The heterogeneity complicates the understanding of disease pathogenesis and poses challenges to the development of effective therapeutic strategies.^1^^,^^12^ Improved understanding of the molecular mechanisms underlying this heterogeneity could therefore facilitate patient stratification and the development of more effective clinical trials and personalised therapeutic interventions.^1^, ^2^, ^3^

To better understand this heterogeneity, large longitudinal studies with harmonised clinical phenotyping and biosampling are required. The INNODIA consortium was established as a European platform for standardised recruitment and follow-up of individuals with newly diagnosed T1D and unaffected family members, with the aim of improving prediction, staging, and evaluation of disease progression.^13^^,^^14^ The INNODIA Natural History Study provides a large multicentre longitudinal cohort of individuals, including children, adolescents and adults with recent-onset T1D, enabling investigation of disease heterogeneity across age groups and over time.^14^

As part of the INNODIA study, we recently showed that gene expression changes in peripheral blood between baseline and one year post-diagnosis were associated with the rate of C-peptide decline at two years, when analysing samples from the cohort of first 100 patients newly diagnosed with T1D (First ND).^15^ These findings highlighted the potential of identifying transcriptomic changes as early indicators of the rate of disease progression. However, larger cohorts of newly diagnosed patients with T1D are essential to confirm the robustness of the findings.

In the present study, we aimed to validate and extend our earlier observations by analysing an independent follow-up cohort of 168 patients newly diagnosed with T1D from the INNODIA study (Next ND). We performed transcriptome profiling of peripheral blood samples collected at diagnosis and at 1-year follow-up and integrated these data with the data from the previously analysed First ND cohort. By combining data from both cohorts, we sought to increase the sample size and statistical power, enabling more comprehensive characterisation of gene expression changes during the first year after T1D onset and their association with the rate of disease progression, to inform future strategies for biomarker development and personalised intervention in T1D.

## Methods

### Ethics

The study followed the guidelines of the Declaration of Helsinki for research on human participants. The study protocol was initially approved by the London–City & East Research Ethics Committee (REC 16/LO/1750; IRAS Project ID 210497). Following translation of the participant documentation, approval was also obtained from the relevant local ethics authorities at the participating study sites. Written informed consent was obtained from all adult participants and from parents or legal guardians of minors. In addition, age-appropriate assent was obtained from underage participants, who were asked to provide their own consent once they reached the age at which this was applicable.

### Clinical cohort

Whole-blood samples were collected as part of the INNODIA study, following a Master Protocol,^13^ at baseline (within 6 weeks of diagnosis) and at 1 year post-diagnosis. This study included 139 samples from the First ND cohort (47 males, 46 females), with a median age of 11.0 years at diagnosis (range 1–38 years), and 317 samples from the Next ND cohort (103 males, 65 females), with a median age of 10.5 years at diagnosis (range 1–42 years). In total, 46 and 149 individuals had samples from both baseline and 1-year follow-up visits from the First ND and Next ND cohorts, respectively.

Sex was recorded as part of the clinical metadata collected at enrolment in the INNODIA study; it was included as a covariate in the statistical analyses where appropriate.

Fasted C-peptide/glucose ratio was used as a proxy to measure disease progression, because mixed meal tolerance test (MMTT) data was not available for participants aged 5 years or younger, greatly limiting the number of participants that could be included in the analyses. C-peptide corrected for glucose has been suggested as a suitable surrogate of MMTT AUC.^15^^,^^16^ Additional clinical characteristics are provided in Table 1, including HbA1c at baseline and 1-year follow-up, BMI at baseline, and autoantibody positivity at baseline.

**Table 1:** Clinical characteristics of samples used in the study.

	First ND	Next ND	Combined
Patients	93	168	261
RNA-seq profiles			
Baseline	93	158	251
1-year	46	159	205
Sex			
Female	46	65	111
Male	47	103	150
Baseline			
IAA			
Positive	70	132	202
Negative	23	35	58
n/a	0	1	1
GAD65			
Positive	73	129	202
Negative	20	38	58
n/a	0	1	1
ZnT8A			
Positive	62	119	181
Negative	31	48	79
n/a	0	1	1
IA-2A			
Positive	64	133	197
Negative	29	34	63
n/a	0	1	1
Age			
Mean	13.1	12.0	12.4
SD	8.5	7.6	7.9
BMI			
Mean	18.9	18.8	18.8
SD	3.9	3.3	3.4
HbA1c			
Mean	79.3	76.2	76.8
SD	22.1	19.3	19.9
Fasted cpep/glucose			
Mean	38.5	40.1	39.7
SD	31.8	28.6	29.3
1-year			
HbA1c			
Mean	54.7	53.4	63.7
SD	14.4	10.3	11.3
Fasted cpep/glucose			
Mean	34.5	33.9	34.0
SD	30.9	29.0	29.4

### RNA-sequencing

Prior to RNA extraction, frozen whole-blood PAXgene samples were thawed at room temperature for 2 h and subjected to RNA extraction using PAXgene Blood miRNA Kit (PreAnalytix/QIAGEN, Cat# 763134). Total RNA, including RNA longer than approximately 18 nucleotides, was purified, following the protocol supplied by the kit manufacturer. Sample concentration was measured with Nanodrop 2000 spectrophotometer and Qubit Fluorometric Quantitation (Thermo Fisher Scientific). The quality of the samples was ensured with Experion Automated Electrophoresis System (Bio Rad) and Agilent 2100 Bioanalyzer RNA Pico chip. Library preparation and sequencing were carried out at the Finnish Functional Genomics Centre (FFGC). Before starting library preparation, ERCC Spike-in Mix 1 (Invitrogen P/N 4456739) was added to 100 ng RNA according to the kit's protocol. RNA-sequencing libraries were prepared using TruSeq stranded mRNA HT kit and protocol #15031047 (Illumina). The quality and quantity of the amplified libraries were measured using Advanced Analytical Fragment Analyser (Agilent) and Qubit Fluorometric Quantitation, respectively. Pooled libraries were sequenced on an Illumina NovaSeq 6000 instrument, using 2 × 50 bp paired-end sequencing.

### Preprocessing of RNA-sequencing data

RNA-sequencing reads were aligned to the human reference genome (hg38) using the Rsubread package (v.2.6.4) which was also used to generate the gene-level read counts based on RefSeq gene annotations. Three haemoglobin-related genes (HBA1, HBA2, HBB) were overrepresented in the data, accounting for around 11.6% of all reads. These reads were filtered prior to normalisation. The filtered count data were normalised using the trimmed mean of the M-values (TMM), converted to counts per million (CPM), and log_2_-transformed using the R package edgeR (v.4.6.3).^17^ Genes with mean logarithmic CPM <1 across the samples were removed to filter out genes with low expression. Principal component analysis (PCA) was performed as a quality control step to evaluate global transcriptomic variation, identify potential outliers, and assess possible cohort-related batch effects.

### Estimation of relative cell type abundances

We quantified relative cell type abundances for seven major cell populations in whole blood using marker-based estimates, including T cells, B cells, plasmablasts, monocytes, neutrophils, erythrocytes, and platelets. For each cell type, 5–6 cell type-specific markers were selected from literature and their cell type-specific expression was confirmed using the CZ CELLxGENE single cell resource,^18^ covering ∼5.6 million cells from healthy human blood. Additionally, we confirmed that the markers for each cell type were highly correlated across the samples in our RNA-sequencing data. The mean value of the cell type-specific markers per sample was used as a proxy for the relative abundance of each cell type. The accuracy of the estimates was confirmed by comparing them with measured cell type proportions using samples with both RNA-sequencing and flow cytometry data and Pearson and Spearman correlation.

### Statistical analysis of changes between baseline and 1-year follow-up

Linear mixed effects modelling was used to identify significant transcriptional changes between samples collected at diagnosis and 1-year follow-up. A separate model was fitted for each gene and cell type, with gene expression level or the relative cell type abundance as the dependent variable, respectively. Visit, age, sex, and sequencing pool (batch) were treated as fixed effects, while study site and individual nested within study site were included as random effects. There were no missing values in the clinical variables used as covariates in the models. The same modelling approach was applied separately to First ND and Next ND cohorts, as well as to the combined dataset. To account for variation in cellular composition in the whole blood RNA-sequencing data, we further included the estimated relative cell type abundances as additional fixed effects in the gene-wise mixed effects models for the combined dataset. The gene-wise linear mixed effects model can be written as

$$y_{ij}=\beta_{0}\;+\;\beta_{1}{Visit}_{ij}\;+\;\beta_{2}{Age}_{i}\;+\;\beta_{3}{Sex}_{i}\;+\;\beta_{4}{Batch}_{ij}+\;\sum_{k}{\gamma kCelltype}_{kij}\;+\;b_{0,site}\;+\;b_{0,i\left(site\right)}\;+\;\varepsilon_{ij}$$

where *y*_*i*j_ denotes the expression level of a given gene for individual *i* at visit *j*. For analyses of estimated relative cell type abundances, the same model structure was used without the cell type covariates. The analyses were performed using the R package lmerTest (v.3.1-3). For the gene-wise analyses, the distribution of nominal p-values was assessed using histogram, quantile–quantile (QQ) plot, and genomic inflation factor (λ). To account for multiple testing across genes, p-values were adjusted using the Benjamini-Hochberg method. Reported p-values refer to nominal p-values unless otherwise indicated, whereas p-values adjusted for multiple testing are reported as false discovery rate (FDR) values.

### Statistical analysis of disease progression rate based on C-peptide/glucose ratios

Similar to our earlier study,^15^ we used the rate of decline in the fasted C-peptide/glucose ratio as a surrogate marker of disease progression, reflecting residual β-cell function. For each individual, we estimated the trajectory of the log-transformed C-peptide/glucose ratio over follow-up visits using linear regression. To ensure robust estimates, we excluded measurements collected at the 3-month visit post-diagnosis to avoid confounding by the so-called honeymoon phase when transient recovery of β-cell function may happen.^19^^,^^20^ Based on the fitted individual-specific trajectory, we then estimated the time at which the predicted logarithmic C-peptide/glucose ratio reached the prespecified threshold of zero. This estimated time was used as the event time in the Cox proportional hazards modelling in a time-to-threshold fashion. Individuals whose predicted trajectory did not reach this threshold within 30 years were censored.

To assess the association between changes in relative abundances of different cell types from baseline to the 1-year follow-up and the rate of disease progression, univariable and multivariable Cox analyses were performed, with the latter adjusting for age and sex. The proportional hazards assumption was evaluated using Schoenfeld residuals and tests for time-dependent effects for the model covariates.

To assess the association between gene expression changes and the rate of disease progression, Cox models were considered at two levels. First, gene-wise models were constructed using within-individual changes in gene expression between diagnosis and 1-year follow-up, adjusted for changes in relative cell type abundances, age at diagnosis, and sex. For each gene, statistical significance was determined using the likelihood ratio test by comparing the model including the gene to a model excluding it. This gene-wise direct change Cox model can be written in the form.

$$h_{i}\left(t\right)=h_{0}\left(t\right)\exp\left(\beta_{1}\Delta y_{i}+\beta_{2}{Age}_{i}+\beta_{3}{Sex}_{i}+\sum_{k}{\gamma k\Delta Celltype}_{ki}\right)$$

where $\Delta y_{i}$ denotes the within-individual change in expression of a given gene for individual *i* and ${\Delta Celltype}_{ki}$ the corresponding change in the estimated relative abundance of cell type *k*. Second, we considered within-individual changes in gene expression residuals derived from linear mixed effects models adjusted for age, sex, batch, site, and relative cell type abundances, and utilised the reproducibility-optimisation approach (ROTS) for the analysis. This gene-wise residual-based Cox model can be written in the form.

$$h_{i}\left(t\right)=h_{0}\left(t\right)\exp\left(\beta_{1}{\Delta r}_{i}\right)$$

where ${\Delta r}_{i}$ denotes the within-individual change in expression residuals of a given gene for individual *i*. The analyses were performed using the R packages survival (v.3.8-3) and ROTS (v.2.0.0), respectively. For the gene-wise analyses, the distribution of nominal p-values was assessed using histograms, QQ plots, and genomic inflation factors. Reported p-values refer to nominal p-values unless otherwise indicated.

The expression scores based on the identified 47 genes were determined as the difference between the mean expression of genes with a hazard ratio <0.5 and the mean expression of genes with a hazard ratio >2, following a similar approach as in.^21^

For Kaplan–Meier analyses, the first and third quartiles were considered. The log-rank test was used to assess the significance of differences between the groups.

### Gene set enrichment analysis

Ranked gene set enrichment analysis against MSigDB (v2023.2) was performed using the R package fgsea (v1.34.2). Genes were ranked based on the formula (1 − p) ∗ sign (coefficient). Here p refers to the p-value from the linear mixed effects or Cox proportional hazards model for a gene, which is multiplied by the sign of the corresponding effect for that gene. To assess whether glycolysis-related expression changes might reflect glycaemic state, the gene-wise mixed effects models for the combined dataset were additionally adjusted for HbA1c and gene set enrichment analysis was repeated using the updated gene ranking. To account for multiple testing, p-values were adjusted using the Benjamini-Hochberg method.

### Role of funders

The funders had no role in the study design, data collection, analysis and interpretation of data, in the writing of the article, or in the decision to submit the paper for publication.

## Results

### Transcriptome changes during type 1 diabetes progression validated in the follow-up cohort

**Fig. 1:**
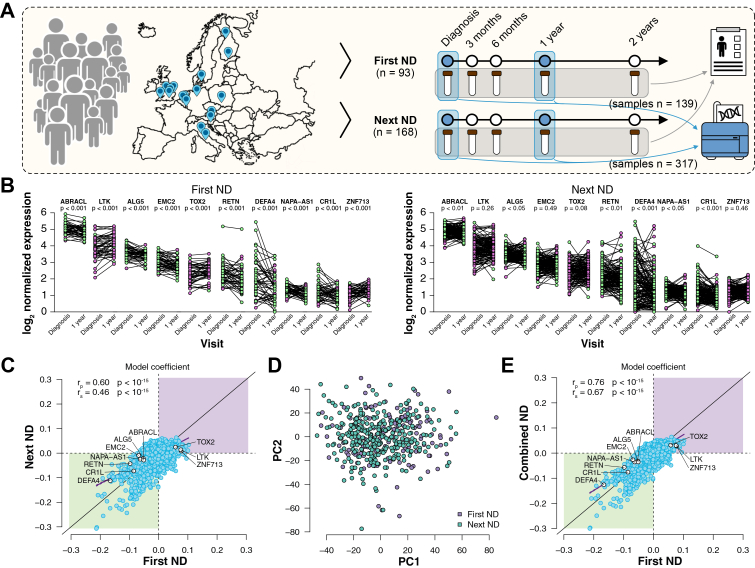
Comparison of transcriptome changes during type 1 diabetes progression between the First ND (93 patients) and Next ND (168 patients) cohorts. **(A)** Whole blood samples were collected from newly diagnosed patients as part of the INNODIA study at diagnosis and 1-year follow-up (139 samples from First ND, 317 samples from Next ND cohorts), while clinical variables were available across multiple follow-up visits. **(B)** Gene expression changes between the baseline and 1-year follow-up visits across individuals for the top ten genes from the First ND cohort.^15^ Log-transformed normalised expression levels (counts per million) are shown for paired baseline and 1-year follow-up samples, with values from the same individual connected with lines. The p-values were obtained from linear mixed effects modelling. **(C)** Model coefficients from linear mixed effects modelling for each gene in the First ND and Next ND cohorts, comparing the baseline and 1-year follow-up visits, together with Pearson (r_p_) and Spearman (r_s_) correlation coefficients and their corresponding p-values. The top ten previously identified candidate genes from the First ND cohort are highlighted. **(D)** Principal component analysis of the RNA-sequencing data from the First ND and Next ND cohorts, showing no clear separation by cohort and supporting comparability of the datasets. **(E)** Model coefficients from linear mixed effects modelling for each gene in the First ND and combined cohorts, comparing the baseline and 1-year follow-up visits. The top ten previously identified candidate genes from the First ND cohort are highlighted.

We previously identified gene expression changes during the first year after the disease onset in the First ND cohort from INNODIA.^15^ Here, we carried out RNA-sequencing on whole blood samples collected at diagnosis and at 1-year follow-up visit from the follow-up cohort of 168 patients newly diagnosed with T1D as part of the INNODIA study (Next ND) (Fig. 1A, Supplementary Fig. S1). We applied linear mixed effects modelling to assess the reproducibility of the results from the First ND cohort for the top ten candidates, defined in the previous study by a threshold of p < 0.001 and absolute model coefficient >0.05^15^: *ABRACL*, *LTK*, *ALG5*, *EMC2*, *TOX2*, *RETN*, *DEF4A*, *NAPA-AS1*, *CR1L*, and *ZNF713.* The observed changes in expression levels of these genes demonstrated consistent trends across both the First ND and Next ND cohorts, with six genes reaching statistical significance also in the Next ND cohort (p < 0.05, Fig. 1B). Moreover, effect sizes across all genes were highly concordant between the cohorts (Pearson correlation 0.60, p < 10^−15^; Spearman correlation 0.46, p < 10^−15^), supporting the robustness of the observed transcriptomic changes (Fig. 1C).

To increase the sample size and statistical power of the analysis, we combined data from the First ND and Next ND cohorts. Principal component analysis (PCA) was used as a quality control step to assess the global structure of the transcriptomic data and potential cohort-related batch effects. The PCA showed an even distribution of individuals across the principal components, with no clear clustering by cohort, supporting comparability of the datasets and their suitability for combined analysis (Fig. 1D).

Analysis of the combined dataset revealed that the differentially expressed genes between the baseline and 1-year follow-up visits were predominantly down-regulated over time, consistent with the pattern observed in the First ND cohort (Fig. 1E). The observed changes in the expression levels of the previously identified top ten candidate genes showed again consistent trends in both the First ND and combined cohorts, and effect sizes across all genes were highly concordant (Pearson correlation 0.76, p < 10^−15^; Spearman correlation 0.67, p < 10^−15^; Fig. 1E).

### Adjusting for the cell types revealed new interesting candidates

**Fig. 2:**
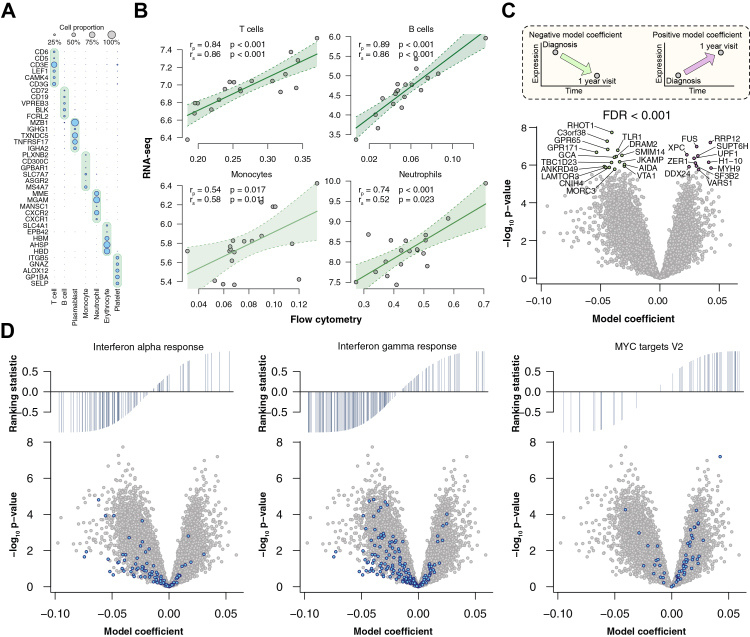
Linear mixed effects modelling while accounting for relative cell type abundances. **(A)** Proportion of cells expressing cell type specific marker genes used for estimating the relative cell type abundances, according to CZ CELLxGENE single cell resource.^18^**(B)** Pearson (r_p_) and Spearman (r_s_) correlations between RNA-sequencing based estimates of relative cell type abundances and flow cytometry based cell type proportions across samples with both measurements available. **(C)** Volcano plot showing the model coefficient from linear mixed effects modelling for each gene, comparing the baseline and 1-year follow-up visits, visualised against their -log_10_ nominal p-value. Genes with false discovery rate (FDR) < 0.001 are highlighted. **(D)** Selected top pathways from ranked gene set enrichment analysis. Genes in the pathway are highlighted.

Due to the complex cellular heterogeneity of whole blood, we next sought to get insight into variation in cell type composition by computationally estimating the relative abundances of circulating cell populations, including T cells, B cells, plasmablasts, monocytes, neutrophils, erythrocytes, and platelets, using cell type-specific marker genes (Fig. 2A). To confirm the accuracy of the estimates, we first compared them with measured cell type proportions using samples with both RNA-sequencing and available flow cytometry data. The estimated and measured proportions showed high correlations for most cell types (Pearson correlation 0.74–0.89, p < 0.001; Spearman correlation 0.52–0.86, p < 0.05; Fig. 2B), supporting the reliability of the computational approach. Monocytes showed a more modest correlation of 0.54 (p = 0.02), in line with recent community benchmarking.^22^

Analysis of the individual cell types revealed significant decreases in the estimated relative abundances of erythrocytes and plasmablasts from baseline to the 1-year follow-up (linear mixed effects model p < 0.001, adjusted for age, sex, and batch; Supplementary Table S1). As expected, the estimated relative abundances of most cell types were significantly associated with age (Supplementary Table S1).

Incorporation of the relative cell type abundance estimates (T cells, B cells, plasmablasts, neutrophils, erythrocytes, platelets) into the linear mixed effects modelling of the transcriptomics data identified a total of 1083 differentially expressed genes at false discovery rate (FDR) < 0.01 between the baseline and 1-year follow-up (Supplementary Table S2), consistent with substantial within-individual transcriptomic changes during the first year after diagnosis. Of these genes, 707 (65%) were down-regulated in the 1-year follow-up and 376 (35%) were up-regulated. The most significantly down-regulated genes were RHOT1, C3orf38, GPR65, TLR1, and GPR171, while the most significantly up-regulated genes included RRP12 and FUS (Fig. 2C). Several of the differentially expressed genes have been associated with T1D in previous studies including the strong candidates PTPN22 and PTPN2,^23^^,^^24^ and also TOX2 (FDR <0.003) identified as a top candidate in our previous study.^15^

Examination of the p-value histogram, QQ plot, and genomic inflation factor (λ = 4.794; Supplementary Fig. S2) showed substantial deviation from the null distribution, which can be expected when a large proportion of genes undergo true expression changes over time.^25^

We further performed ranked gene set enrichment analysis (GSEA) to infer biological functions of the differentially expressed genes. The analysis revealed both down- and up-regulated gene sets during the first year after the disease onset (Supplementary Table S3). Among the down-regulated gene sets, interferon alpha and gamma responses were the most prominent (Fig. 2D). The type I interferon transcriptional signature has been previously associated with the development of T1D.^26^^,^^27^ The most significantly enriched pathways among the up-regulated genes included MYC targets, myogenesis, mitotic spindle, and glycolysis, which are associated with cellular proliferation, differentiation, and metabolism.

To assess whether the glycolysis-related expression changes might reflect glycaemic state, the gene set enrichment analysis was repeated after additional adjustment for HbA1c in the mixed effects models. Notably, these same pathways remained significant, including glycolysis, although the strength of the glycolysis signal was somewhat attenuated (Supplementary Table S3).

### Decreased relative abundance of neutrophils is associated with rapid C-peptide decline

**Fig. 3:**
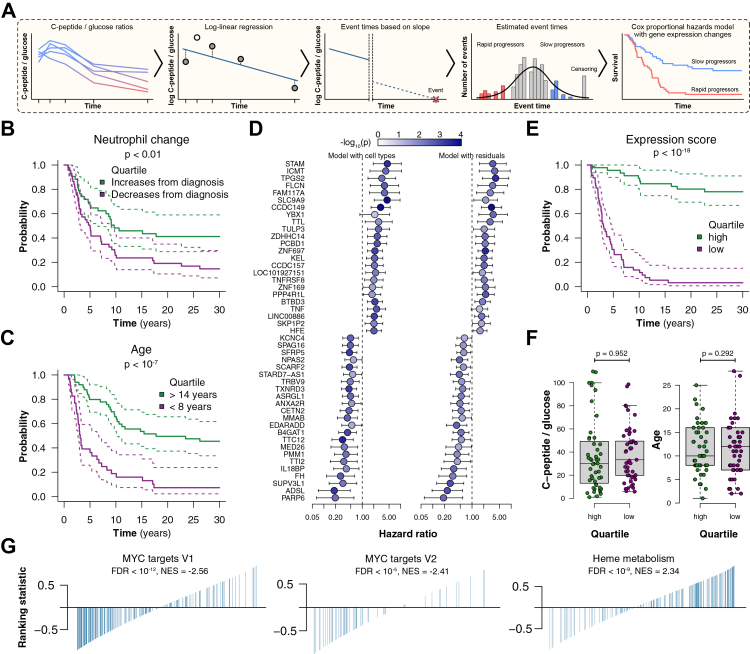
Analysis of gene expression changes associated with rate of disease progression. **(A)** Schematic overview of the analysis approach, using the trajectory of the fasted C-peptide/glucose ratio over time as a surrogate for the rate of disease progression and Cox proportional hazards modelling to determine associations. **(B–C)** Kaplan–Meier curves illustrating the association of decreased abundance of neutrophils (n = 49 in both groups) and younger age (n = 49 for >14 years, n = 47 for <8 years) with more rapid disease progression, respectively. **(D)** Genes whose expression changes from baseline to 1-year follow-up were associated with the rate of disease progression at p < 0.01 and hazard ratio (HR) > 2 or < 0.5 with at least one of the two complementary approaches and supportive evidence from the other (p < 0.1). The model with cell types (left) refers to Cox model using gene expression changes directly, while adjusting for within-individual changes in estimated relative cell type abundances. The model with residuals (right) refers to reproducibility-optimised Cox model using gene expression residuals from linear mixed effects models adjusted for age, sex, batch, site, and relative cell type abundances. Hazard ratios with 95% confidence intervals are shown for each gene. **(E)** Kaplan–Meier curves of the first and third quartiles of expression scores determined based on the identified 47 genes, showing their association with rapid versus slow progression (n = 49 in both groups). **(F)** Box plots of baseline C-peptide/glucose ratios and ages in these two groups based on the first and third quartiles of expression scores (n = 49 in both groups). Between-group comparisons were performed using the Wilcoxon rank sum test. **(G)** Selected top pathways from ranked gene set enrichment analysis.

We next investigated whether changes in gene expression during the first year after T1D diagnosis were associated with the rate of disease progression. As a surrogate measure of β-cell function, we used the fasted C-peptide/glucose ratio, as described earlier.^15^ For each individual, we estimated the trajectory of the log-transformed C-peptide/glucose ratio over time as a measure of disease progression rate and used Cox proportional hazards modelling to determine associations (Fig. 3A). Visual inspection of the individual log-transformed C-peptide/glucose ratio trajectories and residual diagnostic plot supported that a log-linear model provided a reasonable approximation for estimating the disease progression rate (Supplementary Fig. S3), consistent with previous literature.^28^

Univariable analysis of the individual cell types suggested a lower relative abundance of neutrophils and a higher relative abundance of T cells at the 1-year follow-up, compared to baseline, to be associated with a more rapid disease progression (p < 0.05; Supplementary Table S4). After adjusting for age and sex, a lower relative abundance of neutrophils at the 1-year follow-up remained associated with more rapid disease progression (p = 0.05), whereas the other major cell types did not show significant associations (p > 0.1; Supplementary Table S4). Kaplan–Meier analysis of the first and third quartiles of the within-individual changes in the relative neutrophil abundances supported a highly significant association with the rate of disease progression (log-rank test p < 0.01; Fig. 3B). Consistent with previous studies,^29^, ^30^, ^31^, ^32^ younger age was highly significantly associated with more rapid disease progression (p < 10^−7^; Fig. 3C and Supplementary Table S4). Assessment of the proportional hazards assumption indicated evidence of non-proportionality for age (Supplementary Fig. S4). However, since the deviation appeared gradual rather than suggesting a clear alternative model structure based on stratification, age was retained as an important adjustment covariate, while its hazard ratio should be interpreted cautiously as an average effect over follow-up.

### Gene expression changes associated with C-peptide decline

To study gene expression changes associated with the rate of disease progression in a gene-wise manner, we applied Cox proportional hazards models using two complementary approaches. First, we considered within-individual gene expression changes directly while including within-individual changes in the estimated relative cell type abundances in the Cox model to account for compositional cell type changes over time. Second, we considered within-individual changes in gene expression residuals derived from linear mixed effects models adjusted for age, sex, batch, site, and relative cell type abundances using a reproducibility-optimised approach.^33^ Examination of the p-value histograms, QQ plots, and genomic inflation factors (λ = 0.816 for the direct change approach and 0.853 for the residual-based approach; Supplementary Fig. S2) did not indicate substantial deviation from the null distribution, consistent with the more modest effects expected in this setting.

While none of the genes reached statistical significance after correcting for multiple testing (FDR <0.05) with either approach, several showed consistent associations with both approaches, supporting the robustness of these findings. We therefore focused particularly on 47 genes that were identified at nominal p < 0.01 and hazard ratio (HR) > 2 or < 0.5 with at least one approach and had supportive evidence with the other (p < 0.1). Among these, 24 genes (51%) had higher relative expression associated with rapid progression and 23 genes (49%) lower relative expression associated with rapid progression at the 1-year follow-up compared to baseline (Supplementary Table S5, Fig. 3D).

Notably, Kaplan–Meier analysis of the first and third quartiles of expression scores determined based on these 47 genes confirmed their highly significant association with the rate of disease progression (log-rank test p < 10^−18^; Fig. 3E). Interestingly, individuals in the first and third quartiles did not show significant differences in their baseline age nor their baseline C-peptide/glucose ratios (Wilcoxon rank sum test p > 0.2, Fig. 3F). Overall, these results suggest that gene expression changes during the first year after diagnosis may capture molecular signals linked to β-cell decline in addition to compositional changes in circulating cell populations.

Gene set enrichment analysis suggested higher relative expression of haem metabolism and TGFβ signalling, and lower relative expression of MYC targets to be significantly associated with more rapid disease progression concordantly with both approaches (FDR <0.05; Fig. 3G, Supplementary Table S6).

## Discussion

In this study, we investigated gene expression changes associated with T1D progression by analysing RNA-sequencing data from a follow-up cohort of 168 newly diagnosed patients as part of the INNODIA study. The analysis confirmed consistent patterns in the expression of the top ten previously identified genes between baseline and the 1-year follow-up across both cohorts. Further integrated analysis across both cohorts revealed numerous other changes in gene expression during the first year after the disease onset, including down-regulation of genes related to interferon responses. Importantly, we also identified several genes associated with the rate of T1D progression, as well as a significant association of rapid disease progression with decreased relative abundance of neutrophils. Overall, our findings contribute to understanding of gene expression changes during the first year after the diagnosis and their implications for disease progression and treatment.

We observed a decrease of erythrocytes in patients recently diagnosed with T1D during the first year following disease onset. Previous research has reported altered erythrocyte metabolism in patients with T1D,^34^ and hyperglycemia may also contribute to shortened erythrocyte lifespan, potentially explaining the reduced number of the cells. In addition, we detected decreased plasmablast levels in newly diagnosed patients over the same follow-up period. Interestingly, a previous study demonstrated elevated plasmablast frequencies in patients with new-onset T1D compared to non-diabetic and individuals with long-term T1D.^35^ The higher level of plasmablasts in recent onset patients may reflect an active autoimmune response at diagnosis that subsequently diminishes as the disease progresses, leading to lower plasmablast levels during follow-up.

Interestingly, among the genes whose expression level changed during the one follow-up year, we identified several differentially expressed genes that have previously been implicated in T1D pathogenesis, including TYK2, TLR8, PTPN2, PTPN22, ITGB7, all of which harbour SNPs associated with the disease.

We observed that MYC targets and glycolysis-related genes were significantly upregulated in patients with new onset T1D during the one-year follow-up. Notably, several key glycolytic enzymes, including enolase 1 (ENO1), phosphofructokinase (PFKM), pyruvate kinase (PKM), and glucose-6-phosphate isomerase (GPI), were among the upregulated transcripts in immune cells of patients with T1D. These findings may reflect altered metabolic activity during the early post-diagnosis period; however, they should be interpreted cautiously. In the context of recent-onset T1D, changes in metabolic pathway-related gene expression may reflect not only disease progression, but also glycaemic state at sampling, exogenous insulin exposure, and changes in blood cell composition over time. Notably, the pathway-level findings, including glycolysis, remained significant after additional adjustment for HbA1c, supporting that this signal was not explained solely by glycaemic control. In addition, because whole blood comprises multiple immune cell populations with distinct metabolic programmes, pathway enrichment related to glycolysis cannot be directly interpreted as evidence of mechanisms driving β-cell decline. Rather, these findings indicate transcriptomic changes associated with the first year after diagnosis and require a further investigation in more targeted settings.

Next, we examined whether gene expression changes observed during the first year following T1D diagnosis were associated with the rate of disease progression. We found the decrease in neutrophil proportions to be significantly associated with rapid C-peptide loss, which is consistent with our previous study.^15^ Previous studies have shown lower levels of neutrophils in patients newly diagnosed with T1D, as well in autoantibody-positive individuals compared to non-diabetic controls.^36^^,^^37^ A further study confirmed neutropenia in presymptomatic individuals and patients with T1D and demonstrated accumulation of neutrophils in the pancreas in patients with clinical disease and islet autoantibody-positive at-risk subjects.^38^ However, the present study does not allow conclusions about causality, and the observed decrease in circulating neutrophils could either contribute to or result from the disease progression. In particular, it may reflect systemic metabolic dysregulation, insulin deficiency, altered immune cell trafficking, or other processes accompanying early T1D.

Notably, a decrease in circulating neutrophils has also been found to correlate with an increased risk of progression to T1D among autoantibody-positive at-risk individuals.^37^^,^^38^ Additionally, an inverse correlation between peripheral neutrophil levels and the titres of autoantibodies against GAD, IA2, and ZnT8 in individuals with T1D and latent autoimmune diabetes in adults (LADA) has been reported.^39^ Furthermore, individuals who tested positive for all three autoantibodies demonstrated the most significant decrease in neutrophil levels.^39^ The results of our study are in line with previous observations on the important role of neutrophils in T1D and showed that reduction in peripheral neutrophils was associated with rapid loss of β-cell function in patients with clinical disease. Interestingly, a recent study by Sassi et al. identified a neutrophil-enriched blood gene signature associated with teplizumab non-response in both human cohorts (stage 2 and new-onset stage 3) and nonobese diabetic (NOD) mice.^40^

Another aspect of neutrophil involvement in T1D pathogenesis is changes in neutrophil function upon diabetes microenvironment exposure. Interestingly, hyperglycemia affects the neutrophil metabolism, shifting towards a more pro-inflammatory signature and leading to oxidative stress and neutrophil extracellular trap (NET) formation, thereby potentially exacerbating inflammation in T1D. On the other hand, essential functions such as phagocytosis and the oxidative burst, which are critical for effective bacterial clearance, are compromised under conditions of metabolic dysregulation.^41^^,^^42^ This might be reflected in the increased incidence of bacterial infections observed in patients with T1D compared to unaffected controls.^43^^,^^44^

Previous studies have associated more rapid post-diagnosis decline in β-cell function with higher B cell and lower neutrophil immune signatures.^5^ In our study, the association between lower neutrophil abundance and more rapid disease progression was consistent with these earlier observations. However, we did not observe the association of higher B cell gene expression in the present analysis. This difference may reflect variation in study population, including cohort age composition, as well as differences in study design and analyses. In particular, Dufort et al. reported that higher B cell gene expression was associated with more rapid disease progression in younger individuals, but with slower disease progression with older individuals,^5^ while our analyses considered all individuals together. Our findings strengthen previous observation by showing that neutrophil-related changes remain associated with the disease progression also in an independent cohort analysed using a different approach.

Many previous studies have reported a strong correlation between age at diagnosis and the loss of C-peptide, reflecting a more rapid β-cell decline in younger individuals.^29^, ^30^, ^31^, ^32^^,^^45^ The findings of our study align with these earlier observations, indicating that children under the age of 8 years show a significantly more rapid progression compared to those older than 14 years. Moreover, an accumulating body of evidence suggests the existence of age-related phenotypes and potentially distinct mechanisms underlying the pathogenesis of T1D in children diagnosed before the age of 7 years compared to those diagnosed after the age of 13 years.^7^^,^^46^, ^47^, ^48^

Further, our analysis identified 47 genes that showed consistent association with the rate of disease progression. Among these genes, we would like to highlight *TNF*, *TNFRSF8, HFE*, and *STAM*, all of which had higher relative expression in rapid progressors. *TNF* encodes a pro-inflammatory cytokine, whereas *TNFRSF8* is another TNF superfamily member, encoding CD30, which is expressed on activated T and B cells, NK cells, and monocytes. CD30 has been shown to be upregulated in multiple immune-mediated inflammatory diseases^49^ and a relative expansion of a CD30^+^ T cell subpopulation was observed in the peripheral blood of subjects with newly diagnosed T1D.^50^ Thus, the elevated expression of TNF and TNFRSF8 in rapid progressors may suggest increased inflammatory response in the pancreas.

HFE encodes a protein that regulates iron homoeostasis. Beyond iron metabolism, altered HFE has been shown to modulate type I interferon responses during viral infection.^51^ Additionally, HFE variants have been associated with increased risk of late-onset T1D^52^ and maternal HFE genotypes have been associated with increased risk of T1D in the offspring.^52^ Increased expression of the signal transducing adaptor molecule STAM in regulatory T cells has previously been associated with progressively worse future C-peptide decline in patients with newly diagnosed T1D.^53^

In contrast, genes with lower relative expression in rapid progressors may represent anti-inflammatory or protective pathways. Among these, IL18BP encodes an inhibitor of the pro-inflammatory cytokine IL18, and overexpression of IL18BP has been observed to delay hyperglycemia in mice.^54^ SFRP5 has been shown to have anti-inflammatory properties and to support glucose homoeostasis in type 2 diabetes.^55^ Fumarate hydratase (FH), which encodes the enzyme involved in the fumarate metabolism pathway, has been identified as a pivotal regulator of cytokine production. In macrophages, the inhibition of FH results in a reduction of interleukin-10 (IL-10) secretion and an associated increase in TNF levels.^56^ Additionally, the loss of FH contributes to an elevation in interferon β-levels.^56^ Furthermore, lower levels of FH have been observed in the immune cells of patients diagnosed with systemic lupus erythematosus (SLE)^56^ and multiple sclerosis.^57^

One of the main strengths of the present study is that the key findings were evaluated in an independent follow-up cohort. This demonstrates that the observed gene expression changes after diagnosis are reproducible across independent datasets and are not limited to a single cohort. This is particularly important in longitudinal whole-blood transcriptomic studies, where biological heterogeneity between individuals and technical variability can be substantial. This also helps distinguish robust biological signals from cohort-specific findings. In addition, the combined analysis further increased statistical power to characterise progression-associated transcriptomic changes.

Although our findings were validated in an independent follow-up cohort and supported by the increased sample size of the combined analysis, further validation in larger and more diverse cohorts will still be needed, and this study has some other limitations. More detailed metabolic and clinical variables, including detailed MMTT measures, were not available from all study participants, which did not allow additional correlation analyses to be performed with sufficient statistical power. In addition, C-peptide-based measures reflect residual β-cell function rather than exact β-cell mass. Thus, the fasted C-peptide/glucose ratio used here should be interpreted as a functional proxy of disease progression, not as a direct measure of pancreatic β-cell mass.

Because the analyses were based on whole-blood transcriptomic data, mechanistic interpretation should be cautious, as the observed pathway^56^ changes may reflect a mixture of signals from different blood cell populations. An important limitation of whole-blood transcriptomic studies is that differences in bulk gene expression may partly reflect differences in cell type composition. We addressed this by adjusting for estimated relative abundances of major blood cell populations derived through in silico deconvolution, after confirming good agreement with measured cell type proportions from flow cytometry. However, since these estimates are derived from the same bulk transcriptomic data, they are not fully independent of the downstream gene expression values. Although methods for inferring cell type-specific expression or differential expression from bulk data exist, their performance depends on the abundance of the cell populations considered and the specificity of their marker genes.^22^^,^^58^^,^^59^ We therefore considered adjustment for estimated relative cell type abundances to be the most robust and interpretable approach for the present study, while interpreting the findings at the bulk whole-blood level. Finally, the association between lower peripheral neutrophil abundance and more rapid progression is observational and does not establish causality. Reduced neutrophil levels in blood could reflect either a process contributing to disease progression or a consequence of the metabolic and/or immunological changes occurring after diagnosis.

In conclusion, we identified gene expression changes in circulating immune cells of T1D patients during the first year after the diagnosis. Importantly, our results suggest that, in addition to their compositional changes, gene expression changes during the first year of the clinical disease can capture the rate of disease progression and might be helpful in future studies aimed at patient stratification and developing new therapeutics.

## Contributors

INNODIA and INNODIA HARVEST, LO, MP, SøB, AMS, CM provided expertise and facilitated data collection and curation. TS, IS, MK, RL and LLE designed the study. TS, IS, OR, UKK, SyB, LM, EH, TT collected data and performed analyses. TS, IS, OR, RL, LLE wrote the manuscript. TS, IS, OR verified the underlying data. All authors revised the manuscript and approved the final version.

## Data sharing statement

The generated data is person-sensitive and access can be provided by application to the INNODIA Data Access Committee.

## Declaration of interests

MLM has received grants from Breakthrough T1D. MLM reports consulting fees, lecture honoraria, and support for attending the ISPAD Conference 2025 from Sanofi. MLM reports board participation for SAB Bio and a leadership role as Vice-President of INNODIA iVZW. SøB reports lecture honoraria from Sanofi A/S. SøB also held stock in Novo Nordisk A/S, Lundbeck A/S, and Novonesis A/S. CM reports research support paid to KU Leuven by Dexcom, Abbott, Novo Nordisk, and Sanofi. CM also reports that KU Leuven received financial compensation for lectures and other activities from Eli Lilly, Vertex, Roche, Dexcom, Abbott, Medtronic, Novo Nordisk, Insulet, and Sanofi. CM reports that KU Leuven received financial compensation for board participation from Bayer, Biomea Fusion, Boehringer Ingelheim, Eli Lilly, Abbott, Insulet, Medtronic, Novartis, Novo Nordisk, Roche, SAB Bio, Sanofi, and Vertex. CM holds a leadership role as President of the European Diabetes Forum.
