# Supplementary material for: Transcriptomics of type 1 diabetes progression: a validation study in newly diagnosed patients
Source: eBioMedicine. 2026 Jul 7;130:106374. doi: 10.1016/j.ebiom.2026.106374 (PMC13355739; doi:10.1016/j.ebiom.2026.106374)
Supplement: Supplementary Figures [file mmc8.pdf]

Transcriptomics of type 1 diabetes progression: a validation study in newly diagnosed patients

Tomi Suomi[1,2,*], Inna Starskaia[1,2,*], Omid Rasool[1,2], Ubaid Ullah Kalim[1,2], Sylvaine Bruggraber[3], Loredana Marcovecchio[3], Emile Hendricks[3], Lut Overbergh[4], Mark Peakman[5,6], Timothy Tree[6], Søren Brunak[7], Anke M. Schulte[8], Chantal Mathieu[4], Mikael Knip[9,10,11], Riitta Lahesmaa[1,2,12,#], Laura L. Elo[1,2,12,#]

On behalf of the INNODIA Consortium

[1] Turku Bioscience Centre, University of Turku and Åbo Akademi University, FI-20520, Turku, Finland
[2] InFLAMES Research Flagship Center, University of Turku, Turku, Finland
[3] Department of Paediatrics, University of Cambridge, Cambridge, UK
[4] Department of Chronic Diseases and Metabolism, Endocrinology, Katholieke Universiteit Leuven, Leuven, Belgium
[5] Immunology & Inflammation Research Therapeutic Area, Sanofi, MA, USA
[6] Department of Immunobiology, King's College, London, UK
[7] Novo Nordisk Foundation Center for Protein Research, Faculty of Health and Medical Sciences, University of Copenhagen, Copenhagen, Denmark
[8] Sanofi-Aventis Deutschland GmbH, Frankfurt, Germany
[9] Paediatric Research Centre, University of Helsinki and Helsinki University Hospital, Helsinki, Finland
[10] Research Program for Clinical and Molecular Metabolism, Faculty of Medicine, University of 9 Helsinki, Helsinki, Finland
[11] Tampere Centre for Child Health Research, Tampere University Hospital, Tampere, Finland
[12] Institute of Biomedicine, University of Turku, FI-20520, Turku, Finland
* Shared first authors
[#] riitta.lahesmaa@utu.fi, laura.elo@utu.fi

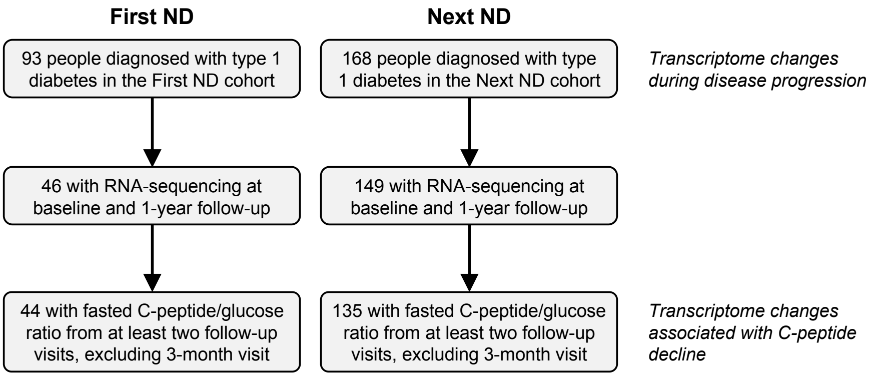

Supplementary Figure 1. Flow chart showing the sample sizes available for the different analyses of transcriptome changes during type 1 diabetes progression in the First ND and Next ND cohorts of the INNODIA study.

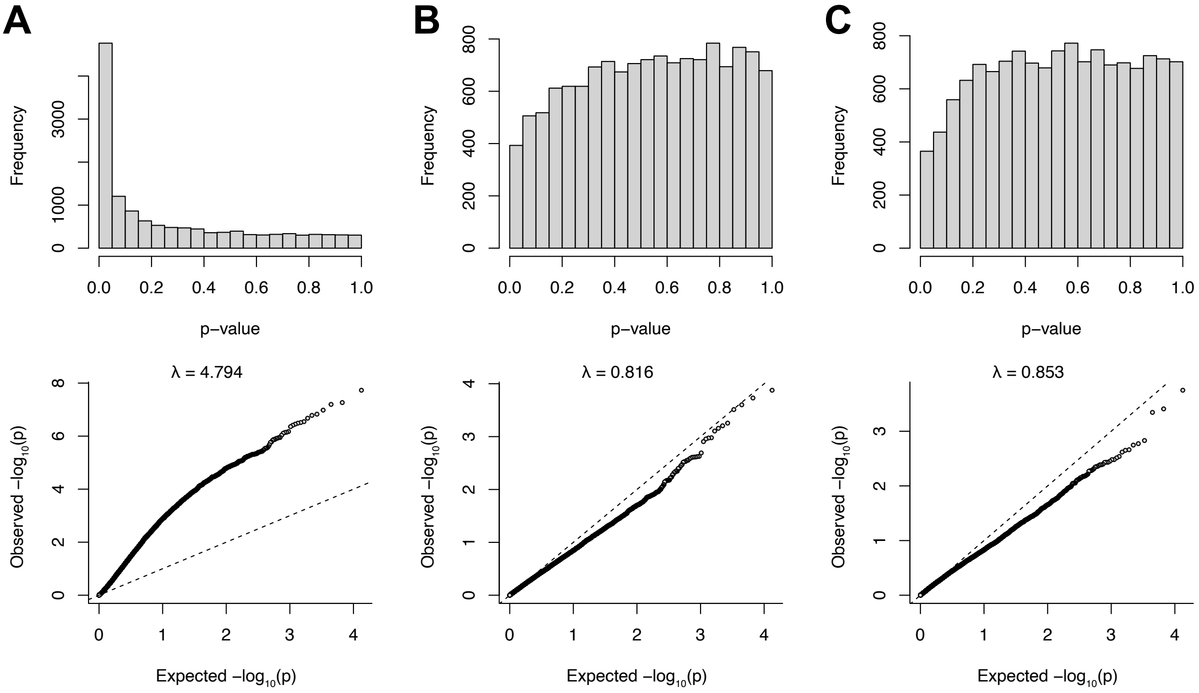

Supplementary Figure 2. Distribution of nominal p-values and the corresponding quantile-quantile (QQ) plots and genomic inflation values (λ) for **(A)** the gene-wise linear mixed effects analysis comparing baseline and 1-year follow-up samples, as well as the gene-wise Cox proportional hazards analyses assessing associations between within-individual transcriptomic changes and the rate of disease progression using **(B)** a direct change approach or **(C)** a residual-based approach.

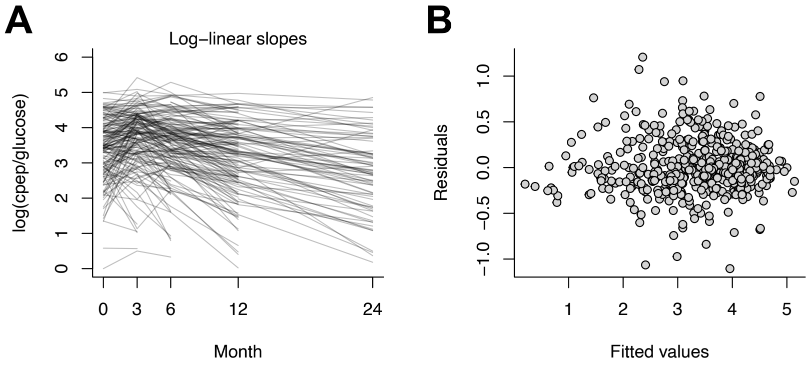

Supplementary Figure 3. Assessment of the log-linear model for fasted C-peptide/glucose ratio over follow-up. **(A)** Individual log-transformed C-peptide/glucose ratio trajectories over time. **(B)** Residuals versus fitted values for the linear regression models.

variable	chisq	p
Age	10.129	0.001
Sex	0.830	0.362
Bcell	0.897	0.343
Erythrocyte	0.158	0.691
Neutrophil	2.229	0.135
Platelet	1.736	0.188
Plasma	1.520	0.218
Monocyte	1.263	0.261
Tcell	1.489	0.222

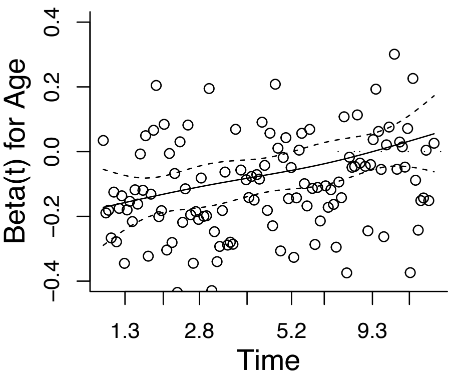

Supplementary Figure 4. Assessment of the proportional hazards assumption in the Cox regression analysis using Schoenfeld residuals. The results indicated evidence of non-proportionality for age (p = 0.001), with the Schoenfeld residual plot suggesting that the effect of age changed gradually with time.
